# Lymphovascular invasion and extranodal tumour extension are risk indicators of breast cancer related lymphoedema: an observational retrospective study with long-term follow-up

**DOI:** 10.1186/s12885-018-4851-2

**Published:** 2018-09-29

**Authors:** Marco Invernizzi, Chiara Corti, Gianluca Lopez, Anna Michelotti, Luca Despini, Donatella Gambini, Daniele Lorenzini, Elena Guerini-Rocco, Stefania Maggi, Marianna Noale, Nicola Fusco

**Affiliations:** 1 Physical and Rehabilitative Medicine, Department of Health Sciences, University of Eastern Piedmont “A. Avogadro”, Viale Piazza D’Armi 1, 28100 Novara, Italy; 2 Division of Pathology, Fondazione IRCCS Ca’ Granda, Ospedale Maggiore Policlinico, Via Francesco Sforza 35, 20122 Milan, Italy; 3 School of Medicine, University of Milan, Via Festa del Perdono 7, 20122 Milan, Italy; 4 Division of Breast Surgery, Fondazione IRCCS Ca’ Granda, Ospedale Maggiore Policlinico, Via Francesco Sforza 35, 20122 Milan, Italy; 5 Division of Medical Oncology, Fondazione IRCCS Ca’ Granda, Ospedale Maggiore Policlinico, Via Francesco Sforza 35, 20122 Milan, Italy; 6 School of Pathology, University of Milan, Via Festa del Perdono 7, 20122 Milan, Italy; 7 Department of Pathology, European Institute of Oncology, Via Giuseppe Ripamonti 435, 20141 Milan, Italy; 8 Department of Oncology and Hemato-oncology, University of Milan, Via Commenda 10, 20122 Milan, Italy; 9 National Research Council (CNR), Neuroscience Institute Aging Branch, Via Giustiniani 2, 35128 Padua, Italy; 10 Department of Biomedical, Surgical and Dental Sciences, University of Milan, Via Commenda 10, 20122 Milan, Italy

**Keywords:** Breast cancer related lymphoedema, Breast cancer, Lymphovascular invasion, Extracapsular extension, Axillary lymph nodes dissection

## Abstract

**Background:**

Breast cancer related lymphoedema (BCRL) occurs in a substantial proportion of breast cancer survivors and is a major contributor to patients’ disability. Regrettably, there are no validated predictive biomarkers, diagnostic tools, and strong evidence-supported therapeutic strategies for BCRL. Here, we provide an integrative characterization of a large series of women with node-positive breast cancers and identify new *bona fide* predictors of BCRL occurrence.

**Methods:**

Three hundred thirty-two cases of surgically-treated node-positive breast cancers were retrospectively collected (2–10.2 years of follow-up). Among them, 62 patients developed BCRL. To identify demographic and clinicopathologic features related to BCRL, Fisher’s exact test or Chi-squared test were carried out for categorical variables; the Wilcoxon rank-sum was employed for continuous variables. Factors associated with BCRL occurrence were assessed using a Cox proportional hazards regression model.

**Results:**

*En-bloc* dissection of the axillary lymph nodes but not the type of breast surgery impacted on BCRL development. Most of BCRL patients had a Luminal A-like neoplasm. The median number of lymph nodes involved by metastatic deposits was significantly higher in BCRL compared to the control group (*p* = 0.04). Both peritumoral lymphovascular invasion (LVI) and extranodal extension (ENE) of the metastasis had a negative impact on BCRL-free survival (*p* = 0.01). Specifically, patients with LVI and left side localization harboured 4-fold higher risk of developing BCRL, while right axillary nodes metastases with ENE increased the probability of BCRL compared to ENE-negative patients.

**Conclusions:**

Assessment of LVI and ENE should be integrated with clinical and surgical data to improve BCRL risk stratification.

**Electronic supplementary material:**

The online version of this article (10.1186/s12885-018-4851-2) contains supplementary material, which is available to authorized users.

## Background

Breast cancer related lymphoedema (BCRL) is a secondary lymphoedema of the upper limb that occurs in up to 54% of breast cancer patients after surgery and/or regional nodal irradiation [1, 2]. Its pathogenesis is currently considered the result of a blockage of the lymphatic fluid from the arm and/or breast, leading to lymph retention [3]. Despite BCRL median onset is 14–18 months post-surgery [4–6], this condition represents a lifelong threat to breast cancer survivors and shows high recurrence rates [7]. Due to the impairment of the upper extremities function and increased comorbidities (e.g. skin infections), BCRL often leads to psychophysical frailty and has a detrimental impact on women’s social life, work, and career [5, 8–11]. Regrettably, the few guidelines available for the diagnosis, screening, and risk assessment of BCRL are not widely adopted [12]. As a result, BCRL patients are often managed using vastly heterogeneous Institution-dependent schemes, in contrast with breast cancer standard of care [13, 14].

The impact that BCRL has both on women’s health and on sanitary costs has led to several lines of research and novel clinical approaches for the prevention and treatment of this condition [5, 15–18]. During the past few years, new strategies have been proposed, such as axillary reverse mapping [19], microsurgical techniques (e.g. LYMPHA) [20], and decongestive physical therapies [21]. These methods together with physical exercise, skin care, and risk factors overall reduction, are grouped in the complex decongestive treatment strategy, a multidisciplinary approach for BCRL clinical management [22]. Despite the great efforts that have been made, the high prevalence of BCRL and the low number of individuals who experience complete remission have currently plateaued [5]. This could be due, at least in part, to the lack of detailed knowledge on the biology underpinning this condition.

Given the extremely high incidence of breast cancer worldwide [23], and the increasing number of long-term survivors [13], the reduction of BCRL burden represents an urgent clinical need in women’s healthcare. However, our ability to identify high-risk individuals remains extremely limited in BCRL, given the lack of reliable biomarkers and predictive tools. Furthermore, net of the mechanistic explanations of its pathogenesis, there are no available data in the literature on tumour-specific features related to BCRL. In this scenario, the aim of the current study was to provide a comprehensive clinicopathological characterization of a large series of surgically-treated node-positive breast cancers with long-term follow-up and to identify clinically relevant subclasses of patients at risk of developing BCRL.

## Methods

### Case selection

This study was fully compliant with the local ethical guidelines and granted Institutional Review Board approval. The medical records of the Fondazione IRCCS Ca′ Granda Ospedale Maggiore Policlinico, Milan, Italy were searched for breast cancers patients who underwent surgical procedures involving both the breast and axilla, including lumpectomy, quadrantectomy, and mastectomy (simple, nipple-sparing, skin-sparing, or radical) with sentinel and/or axillary node(s) excision. Three additional cases were collected from the Division of Physical and Rehabilitative Medicine, University of Eastern Piedmont “A. Avogadro”, Italy. Only patients with data on the presence or absence of upper limb lymphoedema, for which all histologic slides were available for review, as well as detailed clinical and > 2 years follow-up data, were included. Patients with very small incisional biopsies (e.g. core needle biopsy) of the tumour and sentinel lymph node, prior breast surgery (including implants), with tumour measuring < 1 mm in greatest dimensions (i.e. pTmi), with a family history of breast or ovarian cancer and/or *BRCA1* or *BRCA2* mutation, current pregnancy or lactation, or who received neoadjuvant therapy were excluded. Patients were anonymized prior to data collection and analysis. Clinical data included body mass index (BMI), menopausal status, metabolic conditions (e.g. diabetes, dyslipidaemia), infections of the urinary tract, gastroenteric system, and respiratory, type of breast and axillary surgery, therapeutic protocols, and BCRL, which was assessed using a semi-quantitative system during the follow-up oncology visits [24, 25]. Specifically, for all patients with macroscopic evidence of BCRL, the arm volume was measured at different levels from the wrist to humeral head using a circumferential tape and compared to the contralateral arm, as previously described [26].

### Histopathologic review

All cases were re-classified and graded following the latest World Health Organization criteria [27] and the Nottingham grading system [28], respectively. Pathological re-staging was performed according to the 8th edition of the AJCC Cancer Staging Manual [29]. As previously described [30], breast cancer intrinsic molecular subtypes were determined by oestrogen receptor (ER), progesterone receptor (PR), Ki67, and Human epidermal growth factor receptor 2 (HER2) status following the 2017 St Gallen International Expert Consensus recommendations [31]. All diagnostic slides comprising the tumours and lymph nodes were retrieved from the archive and reviewed by two independent breast pathologists (EGR and NF). Discordant results were resolved during a dedicated consensus session. Lymphovascular invasion (LVI) was assessed in the peritumoral tissue on haematoxylin and eosin stained sections according to the criteria proposed by Rosen [32] and endorsed by the College of American Pathologists in the 2017 Protocol for the examination of specimens from patients with invasive carcinoma of the breast (v.4.0.0.0, available at www.cap.org/cancerprotocols). Briefly, LVI was defined by the presence of cancer cells within a definite, endothelial-lined space outside the border of the invasive carcinoma, regardless of the vessel type (i.e. blood or lymphatics) [33]. Tumour emboli with the same shape of the vessel-like structure were considered retraction artefacts [32]. Extranodal extension (ENE) of the metastasis, also referred to as extracapsular extension, was defined by the presence of full-thickness lymph node capsular invasion or extension of tumor cells beyond the lymph node capsule [34–37]. No dimensional cut-off values were employed to assess the extranodal extension.

### Statistical analysis

Categorical variables were represented as the number of patients and the corresponding percentage, whereas continuous variables were summarized through the mean and standard deviation (SD) or through the median and the quartiles (Q1, Q3). Normal distributions of continuous variables were tested using the Shapiro-Wilk test. Relationships between the presence of BCRL and the characteristics of the patient population (i.e. demographic and clinical traits, data on treatment, and pathological features) were assessed using Fisher’s exact test or Chi-squared test for categorical variables, while the Wilcoxon rank-sum test was employed for the continuous variables. Cox’s proportional hazard regression analysis was used to identify factors associated with BCRL occurrence. A purposeful selection of covariates was applied as described elsewhere [38]. The proportional hazard assumption was verified considering Schoenfeld’s residuals of the covariates. In the Cox multivariable model employed, the rule of 10 events per factors was relaxed, as previously described [39]. This allowed for the development of an acceptable model encompassing 5–9 events per predictor. The linearity assumption was evaluated for quantitative variables considering an analysis of quartiles [40]. The presence of significant interactions was also assessed. The hazard ratio (HR) and corresponding 95% confidence intervals (CI) were calculated for each predictor. Survival curves were built according to the Kaplan-Meier method and compared using Log-Rank tests [41]. All statistical tests were two-tailed and *p*-values < 0.05 were considered statistically significant. All the analyses were performed using the SAS 9.4 statistical software (SAS Institute, Cary, NC, USA).

## Results

A total 332 patients (age, 26–88 years; median, 60 years) with node-positive (*N* ≥ 1) breast cancers who were subjected to breast surgery between 1998 and 2015 (follow-up time 2–10.2 years) were included in this study. Their demographic and general characteristics are listed in Table 1 and Additional file 1: Table S1. Among them, 62 (18.7%) patients developed BCRL after 0.4–8.6 years, whereas the remaining 270 (81.3%) patients never showed signs of BCRL.

**Table 1:** Demographic data and treatment information of the patients included in this study, according to their breast cancer related lymphoedema status

Side	BCRL			No BCRL			*p*-value
	Left	Right	Total	Left	Right	Total	
	(*n* = 21)	(*n* = 41)	(*n* = 62)	(*n* = 140)	(*n* = 130)	(*n* = 270)	
Age at diagnosis, years, mean ± SD	60.4 ± 12.5	56.6 ± 13.0	57.9 ± 12.8	58.7 ± 13.0	60.4 ± 13.0	59.5 ± 13.0	0.4734
BMI, n (%)^a^							0.5432
Underweight	0	1 (2.4)	1 (1.6)	4 (2.9)	1 (0.8)	5 (1.9)	
Normal weight	14 (66.7)	13 (31.7)	27 (43.6)	66 (47.1)	55 (42.3)	121 (44.8)	
Overweight	3 (14.3)	18 (43.9)	21 (33.9)	26 (18.6)	44 (33.8)	70 (25.9)	
Obesity	4 (19.0)	9 (22.0)	13 (21.0)	44 (31.4)	30 (23.1)	74 (27.4)	
Menopause, n (%)^b^							0.2647
Pre-menopausal	5 (23.8)	16 (39.0)	21 (33.9)	45 (32.1)	34 (26.2)	79 (29.3)	
Peri-menopausal	0	0	0 (0.0)	5 (3.6)	5 (3.8)	10 (3.7)	
Post-menopausal	16 (76.2)	25 (61.0)	41 (66.1)	90 (64.3)	91 (70.0)	181 (67.0)	
Axillary surgery, n (%)							0.0503
Radical lymph node dissection	21 (100)	41 (100)	62 (100)	130 (92.8)	123 (94.6)	253 (93.7)	
Sentinel lymph node dissection	0	0	0	10 (7.2)	7 (5.4)	17 (6.3)	
Radiotherapy, n (%)							0.3536
Breast	11 (52.4)	22 (53.6)	33 (53.2)	76 (54.3)	72 (55.4)	148 (54.8)	
Breast and supraclavicular fossa	1 (4.8)	6 (14.6)	7 (11.3)	7 (5.0)	7 (5.4)	14 (5.2)	
Supraclavicular fossa and chest wall	4 (19.0)	4 (9.8)	8 (12.9)	22 (15.7)	17 (13.1)	39 (14.4)	
No	5 (23.8)	9 (22.0)	14 (22.6)	35 (25.0)	34 (26.1)	69 (25.6)	
Chemotherapy, n (%)							0.0025
Taxane-based protocol	11 (52.4)	27 (65.9)	38 (61.3)	61 (43.6)	40 (30.8)	101 (37.4)	
Other protocols	1 (4.8)	4 (9.7)	5 (8.1)	14 (10.0)	14 (10.8)	28 (10.4)	
No	9 (42.9)	10 (24.4)	19 (30.7)	65 (46.4)	76 (58.4)	141 (52.2)	
Hormone therapy, n (%)							0.0959
Yes	16 (76.2)	34 (82.9)	50 (80.7)	128 (91.4)	111 (85.4)	239 (88.5)	
No	5 (23.8)	7 (17.1)	12 (19.3)	12 (8.6)	19 (14.6)	31 (11.5)	
Trastuzumab, n (%)							0.0140
Yes	2 (9.5)	7 (17.1)	9 (14.5)	7 (5.0)	8 (6.2)	15 (5.6)	
No	19 (90.5)	34 (82.9)	53 (85.5)	133 (95.0)	122 (93.8)	255 (94.4)	

Abbreviations: *BCRL* Breast cancer related lymphoedema, *BMI* Body Mass Index

^a^BMI was stratified using the WHO International Classification of adult underweight, overweight and obesity, as follows: underweight, < 18.5 kg/m^2^; normal weight, 18.5–24.99 kg/m^2^; overweight, 25–29.99 kg/m^2^; obesity, ≥30 kg/m^2^

^b^Menopausal status was defined according to WHO guidelines. Specifically, menopause is recognized to have occurred after 12 consecutive months of amenorrhea, for which there is no other obvious pathological or physiological cause; peri-menopause is defined as the period immediate prior to the menopause - when the endocrinological, biological, and clinical features of approaching menopause commence, for example variability in the menstrual cycle is increased - and the first 12 months after menopause; pre-menopausal status is used to describe the whole of the reproductive period prior to the menopause

### Type of axillary surgery but not breast surgery impacts on BCRL occurrence

Simple, nipple-sparing, skin-sparing, or radical mastectomy was performed in 22 (36%) BCRL and 106 (39%) no-BCRL patients, while 40 (65%) and 164 (61%) women, respectively, underwent quadrantectomy (Table 1). None of the patients included in this study experienced BCRL without a prior full axillary dissection (*p* = 0.05). Most of BCRL patients (*n* = 48, 77%) were subjected to prior radiotherapy, which included irradiation of the residual breast (*n* = 33, 53%), residual breast and supraclavicular fossa (*n* = 7, 11%), and supraclavicular fossa and chest wall (*n* = 8, 13%). No statistically significant correlations between radiotherapy and BCRL occurrence were observed (*p* = 0.4). During that time period, no patients received axillary surgery and axillary radiation. These observations corroborate the notion that the *en bloc* resection of axillary tissue increases the risk of BCRL more than the only sentinel lymph node excision, irrespective of the type of breast surgery.

### Clinicopathological features of breast cancers that developed BCRL

**Fig. 1:**
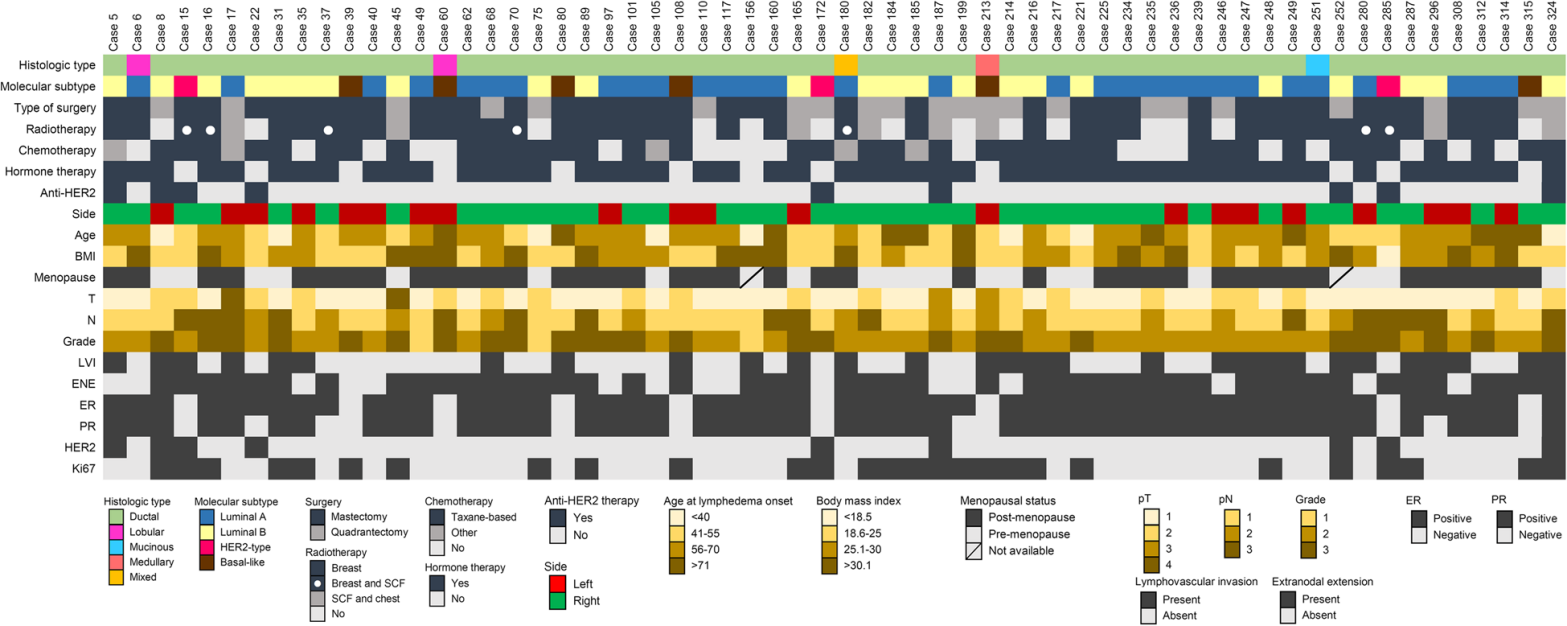
Overview of 62 node-positive breast carcinomas with associated ipsilateral lymphoedema after surgery. Heatmap illustrating the histologic and biological features, surgical, and clinical information. Each column represents a case, each row a parameter, which is color-coded according to the key below. BMI, body mass index; LVI, lymphovascular invasion; ENE, extranodal extension; ER, estrogen receptor; PR, progesterone receptor; SCF, supraclavicular fossa

**Fig. 2:**
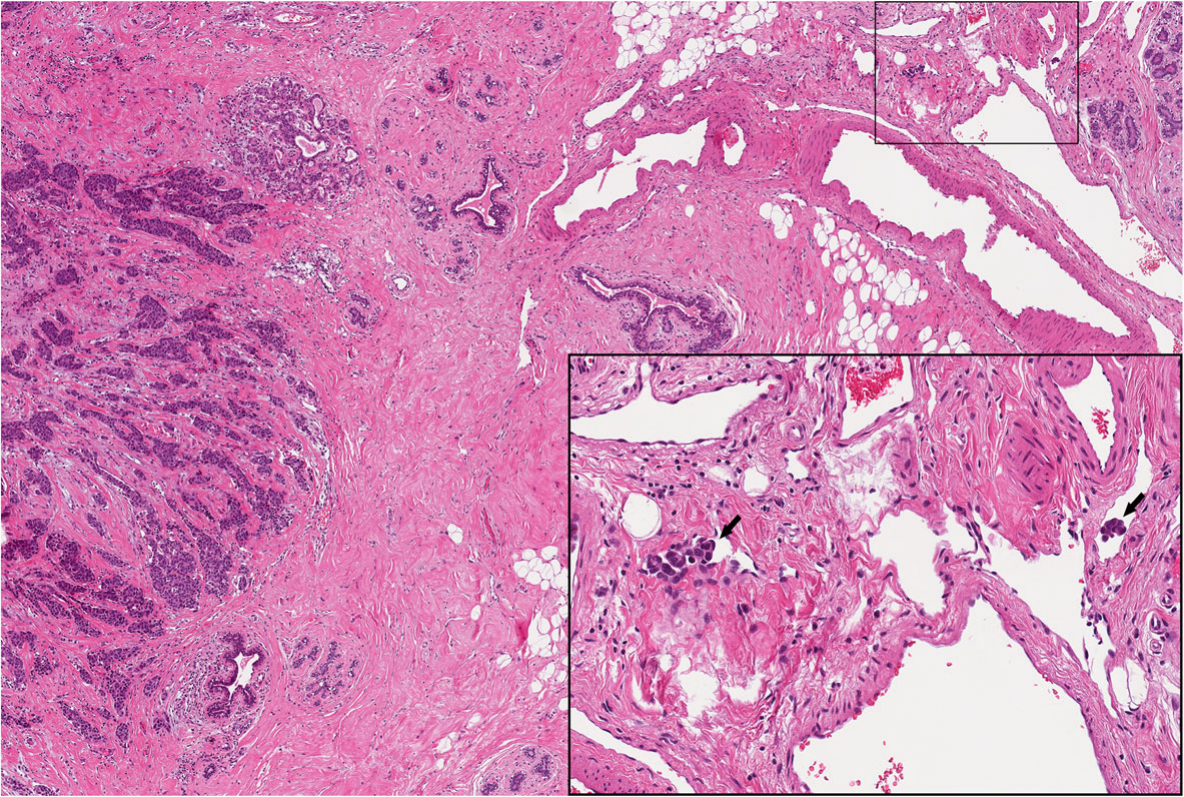
Morphological features of lymphovascular invasion in a patient with breast cancer related lymphoedema after surgery. Representative micrographs of a moderately differentiated invasive carcinoma of no special type showing peritumoral cluster of neoplastic cells inside the lumen of small vessels, as highlighted by the arrows in the inset on the bottom right. One of the two metastatic clusters determined partial lumen obliteration. H&E, original magnification × 100, inset × 400

**Fig. 3:**
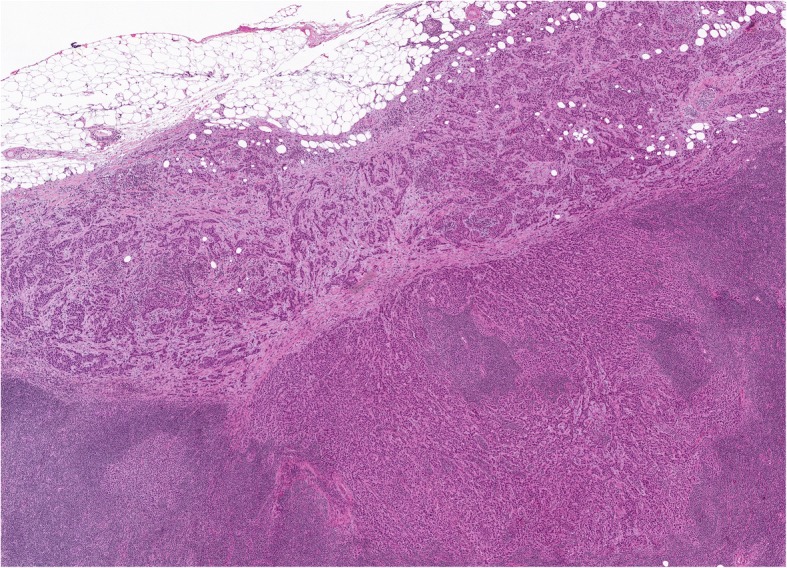
Morphological features of extranodal extension of a lymph node metastasis in a patient with breast cancer related lymphoedema after surgery. Representative micrographs of the axillary lymph node macro-metastasis from a moderately differentiated invasive carcinoma of no special type with extranodal extension to the peri-lymph node adipose tissue. H&E, original magnification × 100

Among BCRL patients (*n* = 62), we observed a significantly high prevalence (*n* = 41, 66%) of late post-surgery BCRL in node-positive breast cancers of the left breast, in contrast to no-BCRL patients (*p* = 0.01), as depicted in Fig. 1 and Table 2. Although the most frequently diagnosed tumour type was the invasive carcinoma of no special type (also known as ductal carcinoma) regardless of the BCRL status, the prevalence of other histological types was lower but not significant in BCRL patients (11% vs. 19%). Most tumours (*n* = 39, 63%) measured less than 2 cm in greatest dimension, being staged as pT1, and were moderately to poorly differentiated (Fig. 1), according to the Nottingham score system [42]. Not surprisingly, most cases were ER positive, PR positive, and HER2-negative (Fig. 1, Table 2). Consistently with the long survival rates that favour BCRL onset, the most frequent molecular subtype (*n* = 30, 48%) was the Luminal A-like (Fig. 1, Table 2). Despite no radiations were administered in the axilla, the median number of lymph nodes involved by metastatic deposits was significantly higher (*p* < 0.05) in BCRL patients (*n* = 3, range 1–7) compared to no-BCRL (*n* = 2, range 1–5), as shown in Table 2. Peritumoral LVI (Fig. 2) was observed in 47% of BCRL, with significantly higher rates (*p* < 0.01) compared to the no-BCRL group (Table 2). Overall, ENE of the lymph node metastasis was identified in 212/332 (63%) tumors (Fig. 3). Among them, the prevalence of ENE-positive cases was higher in the BCRL group (74% vs. 62%, *p* = 0.06). These data suggest that tumour-specific pathologic features are likely to represent risk indicators of BCRL.

**Table 2:** Association between breast cancer related lymphoedema and other clinicopathologic variables

	BCRL	No BCRL	
	(*n* = 62)	(*n* = 270)	*p*-value
Side, left n (%)	41 (66.1)	130 (48.2)	0.0106
Histological type, n (%)			0.1734
NST	55 (88.7)	220 (81.5)	
Others	7 (11.3)	50 (18.5)	
T-staging, n (%)^a^			0.5922
T1	39 (62.9)	157 (58.2)	
T2	19 (30.7)	80 (29.6)	
T3	2 (3.2)	10 (3.7)	
T4	2 (3.2)	23 (8.5)	
N, n (%)^b^			0.1052
N1	33 (53.2)	176 (65.2)	
N2	13 (21.0)	53 (19.6)	
N3	16 (25.8)	41 (15.2)	
G, n (%)^c^			0.6725
1	3 (4.8)	22 (8.2)	
2	33 (53.2)	139 (51.5)	
3	26 (41.9)	109 (40.4)	
ER positive, n (%)	53 (85.5)	241 (89.3)	0.3998
PR positive, n (%)	49 (79.0)	226 (83.7)	0.3791
HER2 positive, n (%)^d^	9 (14.5)	22 (8.2)	0.1202
Ki67 positive, n (%)^e^	28 (45.2)	102 (37.8)	0.2828
Molecular subtype, n (%)			0.6807
Luminal A	30 (48.4)	147 (54.4)	
Luminal B (HER2+)	5 (8.1)	12 (4.4)	
Luminal B (HER2-)	18 (29.0)	82 (30.4)	
HER2-type	3 (4.8)	10 (3.7)	
Basal	6 (9.7)	19 (7.0)	
ENE, n (%)	46 (74.2)	166 (61.5)	0.0603
N. metastatic lymph., median (Q1, Q3)	3 (1, 7)	2 (1, 5)	0.0470
Total n. lymph. Evaluated, median (Q1, Q3)	23 (19, 30)	23 (18, 29)	0.4557
% lymph. Metastatic, median (Q1, Q3)	11.1 (5.6, 31.8)	9.5 (4.5, 25)	0.2062
LVI, n (%)	29 (46.8)	80 (29.6)	0.0095

Abbreviations: *BCRL* breast cancer related lymphoedema, *NST* invasive breast cancer of no special type, *ER* estrogen receptor, *HER2* human epidermal growth factor receptor 2, *ENE* extranodal extension, *LVI* lymphovascular invasion

^a^Tumor dimension (T) according to TNM classification was as follows: T1, Tumor ≤20 mm in greatest dimension; T2, Tumor > 20 mm but ≤50 mm in greatest dimension; T3, Tumor > 50 mm in greatest dimension; T4, Tumor of any size with direct extension to the chest wall and/or to the skin (ulceration or skin nodules)

^b^Pathologic lymph node status (pN) according to TNM classification was as follows: pN0, negative; pN1, 1 to 3 positive lymph nodes; pN2, metastases in 4–9 axillary lymph nodes; pN3, metastases in ≥10 axillary lymph nodes

^c^Grading was established using the Nottingham histologic grading system

^d^HER2 status was assessed using immunochemistry and chromogenic in-situ hybridization in borderline cases

^e^Positivity for Ki67 was defined as ≥10%

### Lymphovascular invasion and extranodal extension increase the risk of BCRL according to the side of surgery

**Fig. 4:**
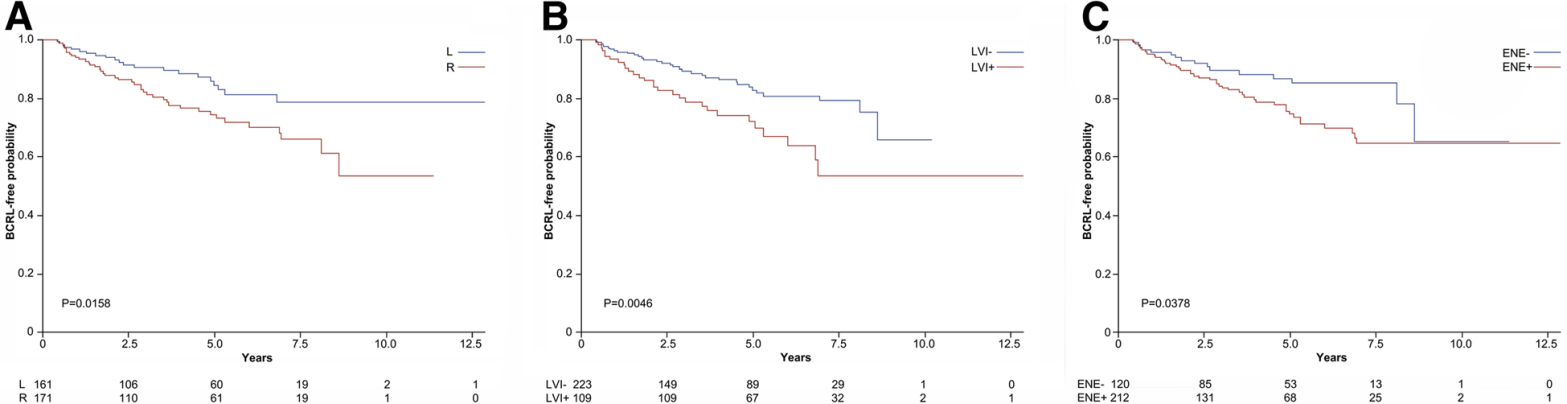
Lymphoedema-free survival of the patients included in the study for selected tumor characteristics. **a**. Probability according to the side of surgery; **b**. Probability according to the presence of lymphovascular invasion; **c**. Probability according to the presence of extranodal extension. The curves were built according to the by Kaplan-Meier method, *p* values are the expression of Log-rank test. The specific risk for a given timeframe is reported on the bottom of each graph. L, left; R, right; LVI+, lymphovascular invasion positive; LVI-, lymphovascular invasion negative; ENE+, extranodal extension positive; ENE-, extranodal extension negative

**Fig. 5:**
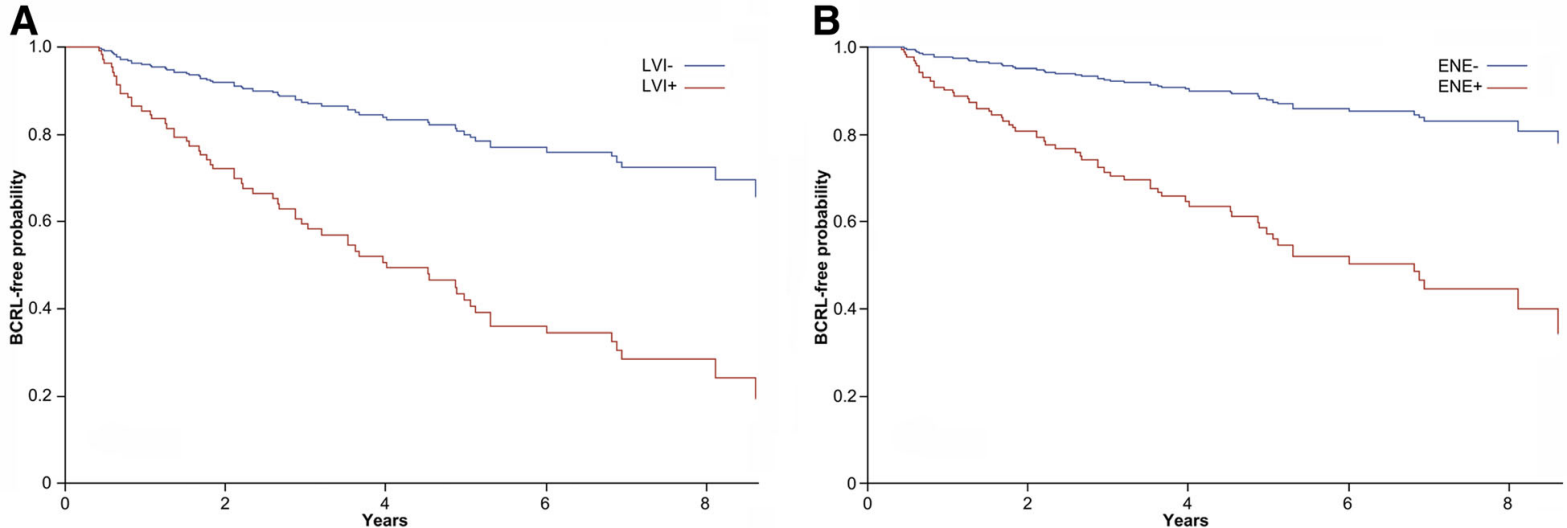
Lymphoedema-free survival of the patients included in the study for selected tumor characteristics on the basis of the side of the surgery. **a**. Probability according to the presence of peritumoral lymphovascular invasion after surgery of the left axilla. **b**. Probability according to the presence of extranodal extension of the lymph node metastasis after surgery of the right axilla. The curves are the result of a Cox proportional hazard regression analysis. LVI+, lymphovascular invasion positive; LVI-, lymphovascular invasion negative; ENE+, extranodal extension positive; ENE-, extranodal extension negative

Log-rank test showed that tumour laterality (*p* < 0.01), peritumoral LVI (*p* < 0.01), and ENE (*p* = 0.04) of the lymph node metastases had a significant impact on BCRL-free survival, as represented in Fig. 4. Analysis of the HR using the Cox regression showed that the presence of LVI represented a significant risk factor within left-side BCRL (HR = 3.78, 95% CI (1.57–9.10)), in contrast to the patients with LVI and right side involved (HR = 1.43, 95% CI (0.75–2.73)), as shown in Table 3 and Fig. 5a. On the other hand, patients who underwent axillary surgery for a node-positive breast cancer located in the right side had more than 3-fold higher risk to suffer BCRL if the macrometastases displayed ENE (HR = 3.38, 95% CI (1.53–7.49)), as shown in Table 3. Interestingly, a similar observation could not be made for the patients with ENE and left-side BCRL (HR = 0.73, 95% CI (0.30–1.78)). In particular, BCRL-free survival was significantly better in right-side breast cancers showing no ENE compared to ENE-positive cases (Fig. 5b). Taken together, the interaction between the side of dissection and ENE resulted statistically significant (*p* = 0.01). On the other hand, there was no difference in BMI, N-stage, and presence of ENE and LVI between patients who were left- or right-handed, as detailed in Additional file 1: Table S1. Consistent with these findings, the interaction between LVI and ENE appeared borderline significant (*p* = 0.07). These data provide evidence consistent with the notion that the routinary assessment of LVI and ENE, that is currently made for prognostic purposes, might be integrated with clinical and surgical data to predict which node-positive breast cancer patients are at higher risk to develop BCRL. Other patients’ clinical conditions significantly associated with the development of BCRL included dyslipidaemia (*p* = 0.04) and post-surgery infections (*p* = 0.05), confirming the prominent role of the clinical milieu in BCRL occurrence. The distribution of right- and left-sided breast cancers according to the presence of ENE and LVI is shown in Additional file 1: Table S2.

**Table 3:** General characteristics of patients and pathological factors associated with the development of BCRL

		HR	95% CI	*p*-value
Infections		2.20	0.99–4.92	0.0540
Dyslipidaemia		0.22	0.05–0.95	0.0431
Body Mass Index (BMI) ≥ 25 kg/m^2^		1.09	0.65–1.84	0.7368
Estrogen receptor positive		13.0	2.31–72.8	0.0036
Progesterone receptor positive		0.44	0.15–1.28	0.1326
Hormone therapy		0.02	0.01–0.09	< 0.0001
Hormone therapy (time-dependent)		2.45	1.16–5.17	0.0187
Side	Extranodal extension			
Right	Yes vs. No	3.38	1.53–7.47	
Left	Yes vs. No	0.74	0.30–1.81	
Side	Lymphovascular invasion			
Right	Yes vs. No	1.41	0.74–2.70	
Left	Yes vs. No	3.80	1.58–9.16	

The hazard ratio (HR) of developing BCRL was calculated using Cox Proportional Hazard Model

## Discussion

BCRL is a relatively frequent condition that, despite being not lethal per se, is extremely detrimental to the quality of life of breast cancer survivors. All breast cancer patients who undergo breast surgery and/or irradiation of the axilla are at risk for lymphoedema, while cases showing complete remission remain rare to date [43]. BCRL shows poor response to surgical, physical, and medical therapies, so guidelines for the management of these women need to be further implemented. Here, we performed a comprehensive clinicopathologic analysis of a large series of surgically-treated node-positive breast cancers with long-term follow-up and found that LVI and ENE have a strong predictive value for BCRL occurrence. Furthermore, to achieve the optimal risk stratification, we documented that the analysis of these two prognostic variables should be integrated with information on the laterality of the tumour and the surgical procedure. Finally, we confirm that the full excision of the axillary nodes is one of the major determinants of BCRL, regardless of the extent of the surgical procedure involving the breast.

The scarcity of literature on BCRL risk indicators is reflected by the absence of clinical nomogram to select patients who would benefit more from tailored anti-lymphoedema surgical and medical interventions. In our study, we observed that women with metastatic breast cancer of the left breast that undergo *en bloc* axillary resection are more likely to develop BCRL compared to patients with right-sided breast cancer. To our knowledge, this is the first time that data on surgery laterality are implicated in BCRL pathogenesis. One of the possible explanations of this observation involves the protective role of physical exercise. Indeed, in contrast to the historical concept that patients after radical lymphadenectomies should avoid physical activity, recent guidelines recommend supervised exercise of the arm to reduce the risk of lymphoedema development [44]. Despite complete data on the dominant arm of our patients were not available, we can posit that the patients included in this study displayed the same prevalence of right-handed individuals than the general population [45]. Following this assumption, up to 95% of the BCRL women were right-dominant. Therefore, our data provide circumstantial evidence to suggest that even the physiological use of the dominant arm has a protective role against BCRL and that women with metastatic breast cancer of the left breast are at higher risk for this condition. Furthermore, we corroborate the notion that even a minimal amount of physical activity has the potential to reduce the likelihood of arm swelling.

Given that no previous study focused on tumour-specific clinicopathological features of the primary tumour to assess BCRL risk, we sought to analyse a pool of histology-based prognostic parameters. Taken together, we observed an overall high frequency of ENE-positive tumors (63%), consistent with previous data on large-scale studies considering any penetration of the lymph node capsule as ENE [35–37]. Hence, this feature shows a high variability in the literature, ranging from 23 to 66% of metastatic breast carcinomas [34, 37, 46–52]. In contrast to LVI, there is currently lack of consensus on how to determine and report ENE, with some groups employing the cut-off value of 2 mm of perpendicular diameter for its assessment [34, 52, 53]. Interestingly, we observed that both LVI and ENE are associated with a poorer outcome in terms of BCRL-free survival when considered in the bivariate analyses. Surprisingly, when the laterality was incorporated in a multivariate model encompassing the hormone receptor status and subsequent hormone therapy, we observed that patients with left-sided breast cancer showing peritumoral LVI have four times higher risk to develop BCRL compared to patients with LVI-negative tumours of the left breast. On the other hand, patients with cancer of the right breast whose metastases showed ENE and that underwent surgery in the homolateral axilla harbour three times higher HR of BCRL in comparison to patients with ENE-negative tumours. No statistically significant interaction between LVI and ENE were observed, therefore no attempt to isolate two distinct populations of patients with either LVI or ENE was made. Notably, the hormone therapy was involved in BCRL occurrence, consistent with the role of ER as modulator of the vascular tropism [54].

The biology that underpins the role of these two prognostic factors based on the side of surgery could be hypothesized using physical algorithms. In particular, the propulsion of the lymphatic fluid in the collecting vessels is strongly affected by preload, afterload, and transmural pressure, as postulated by Frank-Starling law [55]. Shear stresses in addition to nerve and humoral mediators are also implicated in this complex mechanism [56]. Extrinsic stimuli such as skeletal muscle contraction during normal activities, the motion of adjacent organs and even arterial pulsations can also influence the lymphatic flow [56]. Passive flow owing to a positive pressure gradient may also occur in oedema, during which lymph formation and swelling are increased [57]. In murine models, Gashev et al. showed that the intrinsic drainage mechanism is dominant for low levels of lymph formation, but, as these levels arise, the active lymph pump is inhibited, and the vessels become conduits [56, 58]. The different HR of developing BCRL found in a patient with LVI/side-left involved and ENE/side-right involved might be explained on the basis of the mechanisms regulating lymph node propulsion and drainage, since there are not significative anatomical differences between the lymphatic drainage of the two sides of the body [59]. Specifically, the smallest metastatic clusters of cells that are able to determine obstruction to the lymph drainage measure approximately 5–10 μm, which is the average capillary diameter [59]. Therefore, our results provide evidence, albeit circumstantial, to suggest that LVI can represent a physical obstacle to lymph drainage, particularly in small vessels [60]. This phenomenon can become more evident in the left arm, where the muscular pump that typically supports the normal drainage as a *vis a tergo*, is less represented in the non-dominant arm [61]. Furthermore, ENE of the metastatic clones can be physically referred to a breach in the lymph node capsule [56]. Interestingly, lymph nodes present a relatively high resistance to flow [62], while extrinsic mechanisms such as skeletal muscle contraction - a dominant factor if considering that around 90% of world population is right-handed [61] - could be responsible for the continuous compression of those breached lymph nodes. It should be noted, however, that this proposed mechanism has necessarily to take place before surgery and the consequent removal of axillary lymph glands. Still, a latent and subclinical form of lymphoedema could represent a possible scenario even in pre-surgical settings, thus ascribing to surgery an unmasking role by disrupting axillary anatomical structures. This hypothesis is supported by a recent prospective study performed on 1028 breast cancer women, showing that, without baseline (preoperative) evaluation of the arms symmetry, up to 50% of BCRL cases can be missed [63]. Further clinical studies coupled with functional experiments are warranted to explore the complexity of BCRL pathogenesis, also in lights of these novel observations.

This study has several limitations. First, given its retrospective nature, it was not possible to obtain a baseline measurement of the limb prior to surgery and prior to the development of macroscopic BCRL, while the comparison of the two arms might be suboptimal as upper limbs are rarely symmetrical at baseline [63]. Second, all the measurements were not taken at regular intervals but either during routine follow-up visits or after the patient personally contacted the oncologist. Third, mild and asymptomatic forms of BCRL may not always be recorded. These intrinsic limitations could have led to an underestimation of the BCRL incidence in our population of patients. However, this study should be considered hypothesis-generating, as it provides previously unavailable data on pathologic risk indicators of BCRL. Furthermore, there are several lines of evidence that regional nodal irradiation is associated with a significantly higher risk of lymphedema than no irradiation or irradiation of the breast/chest wall after axillary nodes dissection [2]. Hence, recent data (e.g. The European Organisation for Research and Treatment of Cancer (EORTC) and the National Cancer Institute of Canada MA-20 trials) support an increasing role of regional nodal irradiation in the treatment of breast cancer, particularly in early-stage tumors with high-risk features, such as extranodal extension. However, none of our BCRL patients were subjected to axillary radiotherapy, given that they were all surgically-treated in a timeframe ranging from 1998 and 2015. For this reason, it was not possible perform correlations between axillary radiation and BCRL occurrence. On the other hand, we confirm that irradiation of the residual breast, supraclavicular fossa, and/or chest wall should not be considered as risk factors for BCRL development. Further large-scale prospective studies, coupled with standardized BCRL scoring systems, are warranted to define the clinical impact of our findings. Despite these limitations, our work paves the way to a novel tailored clinical approach to BCRL, where an integrative screening platform taking into account clinicopathologic variables together with surgical information could be pragmatically employed on pre-surgical core biopsies and sentinel lymph node procedure. Furthermore, the present study provides novel insights for the set-up of future prospective studies to identify and understand the molecular basis of BCRL with the integration of intrinsic prognostic and predictive biomarkers.

## Conclusion

This study represents the first analysis of BCRL to provide preliminary data of bona fide tumour-specific risk indicators, such as laterality of the surgery and the presence of LVI and ENE. Our observations suggest that the routinary evaluation of LVI and ENE either on pre-surgical (e.g. core biopsies), intra-surgical (e.g. intraoperative sentinel lymph nodes), or post-surgical (e.g. breast and axillary nodes excision) samples might represent the basis for a novel strategy in the identification of patients at higher risk of BCRL. This could have tremendous implications for BCRL management, leading to the development of innovative tailored treatment protocols, even at pre-surgery level.

## Additional file

Additional file 1: **Table S1**. Selected clinicopathologic features of the patients included in this study according to the side of surgery. **Table S2**. Extranodal extension and lymphovascular invasion subgroups of breast cancers according to the side of surgery. **Figure S1**. Lymphedema-free survival of the patients included in the study for selected tumor characteristics on the basis of the side of the surgery. (PDF 433 kb)

## Abbreviations

BCRL: Breast cancer related lymphoedema
BMI: Body mass index
CI: Confidence interval
ENE: Extranodal extension
ER: Oestrogen receptor
HER2: Human epidermal growth factor receptor 2
HR: Hazard ratio
LVI: Lymphovascular invasion
PR: Progesterone receptor
SD: Standard deviation
