# Supplementary material for: Lymphovascular invasion and extranodal tumour extension are risk indicators of breast cancer related lymphoedema: an observational retrospective study with long-term follow-up
Source: BMC Cancer. 2018 Sep 29;18:935. doi: 10.1186/s12885-018-4851-2 (PMC6162920; doi:10.1186/s12885-018-4851-2)

Additional file 1
Table S1. Selected clinicopathological features of the patients included in this study according to the side of surgery.

	Side		Total
	Left (n=161)	Right (n=171)	(n=332)
BMI ≥ 25 kg/m^2, n (%)	79 (49.1)	84 (49.1)	163 (49.1)
ENE, n (%)	104 (64.6)	108 (63.2)	212 (63.9)
LVI, n (%)	52 (32.3)	57 (33.3)	109 (32.8)
N, n (%)			
N1	99 (61.5)	110 (64.3)	209 (62.9)
N2	34 (21.1)	32 (18.7)	66 (19.9)
N3	28 (17.4)	29 (17.0)	57 (17.2)

Table S2. Extranodal extension and lymphovascular invasion subgroups of breast cancers according to the side of surgery.

	Side				Total	
	Left (n=161)		Right (n=171)		(n=332)	
	Num*	Den§	Num*	Den§	Num*	Den§
ENE+ LVI+	8	35	15	37	23	72
ENE- LVI+	4	17	2	20	6	37
ENE+ LVI-	5	69	18	71	23	140
ENE- LVI-	4	40	6	43	10	83

*: number of patients with BCRL in each subgroup (numerator)
§: number of patients in these subgroups (denominator)

Figure S1. Lymphedema-free survival of the patients included in the study for selected tumor characteristics on the basis of the side of the surgery. A. probability according to the presence of peritumoral lymphovascular invasion and side of the surgery. B. probability according to the presence of extranodal extension of the lymph node metastasis and side of the surgery. The curves were built according to the by Kaplan-Meier method, p values are the expression of Log-rank test. The specific risk for a given timeframe is reported on the bottom of each graph. LVI+, lymphovascular invasion positive; LVI-, lymphovascular invasion negative; ENE+, extranodal extension positive; ENE-, extranodal extension negative.

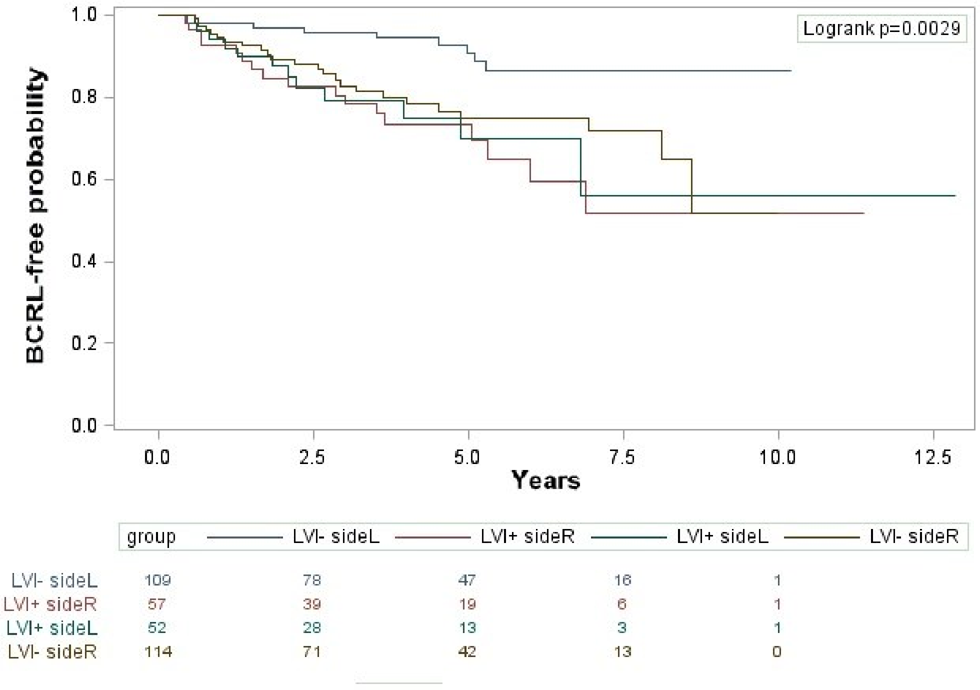

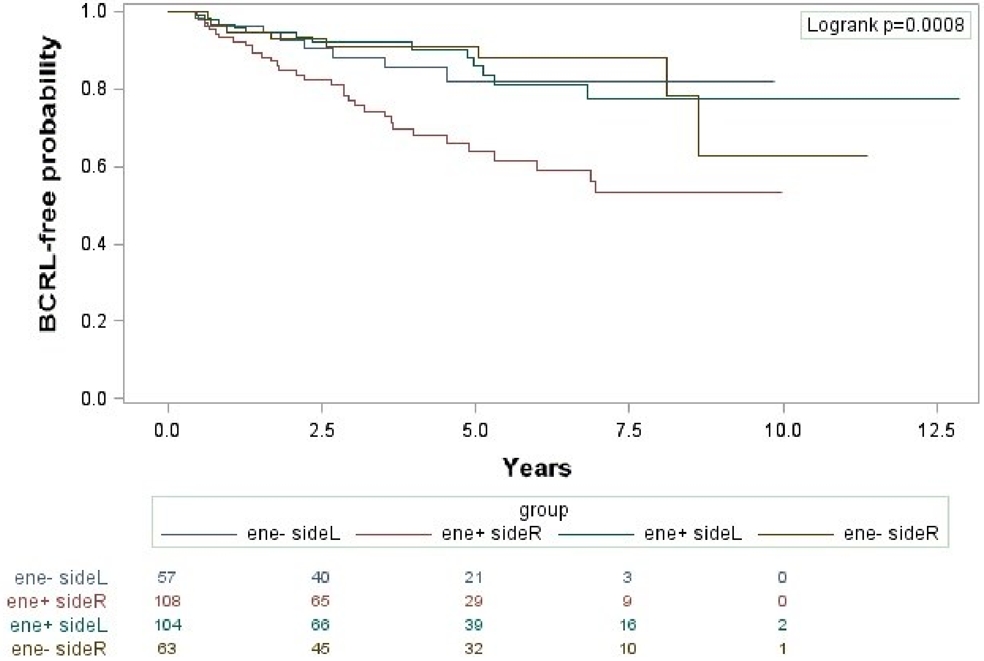

Supplement: Supplementary file 1 — Table S1. Selected clinicopathologic features of the patients included in this study according to the side of surgery. Table S2. Extranodal extension and lymphovascular invasion subgroups of breast cancers according to the side of surgery. Figure S1. Lymphedema-free survival of the patients included in the study for selected tumor characteristics on the basis of the side of the surgery. (PDF 433 kb) [file 12885_2018_4851_MOESM1_ESM.pdf]
